# Posterior Reversible Encephalopathy Occurring During Treatment With Palbociclib

**DOI:** 10.7759/cureus.16604

**Published:** 2021-07-24

**Authors:** Lahcene Belaidi, Nabil Baba-Hamed, Francesco Savinelli, Eric Raymond

**Affiliations:** 1 Department of Oncology, Saint-Joseph Hospital, Paris, FRA

**Keywords:** palbociclib, ibrance, cdk inhibitor, pres, posterior reversible encephalopathy syndrome

## Abstract

Palbociclib (Ibrance™) has been marketed since 2015 for patients with metastatic hormone-receptor-positive breast cancer. We report here the case of a patient who presented with a posterior reversible encephalopathy syndrome (PRES) during treatment with this new targeted therapy.

The 67-year-old woman presented prodromal headaches followed by occurrences of two episodes of generalized convulsive seizures. The brain MRI revealed a bilateral, globally symmetrical, sub-cortical parietooccipital fluid-attenuated inversion recovery (FLAIR) hypersignal of the white matter.

The patient recovered after palbociclib discontinuation with no further neurological signs. A follow-up MRI performed one month upon palbociclib discontinuation showed a decrease in the FLAIR signal abnormalities. Altogether, the clinical presentation was consistent with PRES.

This case report aims to encourage physicians whom patients are treated with cyclin-dependent kinase 4/6 inhibitors to cautiously monitor symptoms suggesting PRES in contexts known to promote its occurrence such as that of arterial hypertension, immunosuppression, and/or autoimmune disease. PRES should be considered in the event of seizure, headache, and/or visual disturbances.

## Introduction

Palbociclib (Ibrance™) is a cyclin-dependent kinase 4/6 inhibitor that promotes G1 cell cycle arrest and inhibits tumor growth. It is registered in combination with letrozole since 2015 as first-line therapy for patients with metastatic hormone-receptor-positive breast cancer [1].

In clinical trials, palbociclib was shown to be associated with multiple side effects such as hematological toxicity (anemia, leukopenia, and thrombocytopenia), fatigue, nausea, and alopecia [1]. However, due to the limited number of patients entering clinical trials, a prospective analysis of side effects may be limited in capturing events of low frequency. Post-marketing pharmacovigilance for marketed drugs has been recognized as an important way of identifying rare and unexpected toxicities.

The high incidence of breast cancer (in the U.S, it is estimated that 150,000 - 250,000 women are currently living with metastatic breast cancer [2]) and the sustained activity of palbociclib/letrozole may be assumed to be associated with an increasing number of exposed women over years to come. Thereby, adverse events of low frequency are likely to be progressively identified and recognized early to optimize patient care.

Posterior reversible encephalopathy syndrome (PRES) is a clinical and radiological entity characterized by headaches, seizures, altered mental status, and visual loss, specified by white matter vasogenic edema affecting the occipital and posterior parietal lobes of the brain predominantly. Hypertension, immunosuppression, and autoimmune diseases are predisposing factors [3]. Symptoms are usually reversible although cases of permanent neurological deficits have already been described [4].

Herein, we report the case of a patient who presented with PRES during treatment with palbociclib.

## Case presentation

A 67-year-old woman had been treated for two occurrences of breast cancer, one in 2003 and another in 2010, both requiring surgical resection and radiotherapy. In July 2020, back pains revealed multiple bone metastasis. First-line therapy with palbociclib (125 mg once daily for 21 consecutive days followed by seven days off treatment) in combination with letrozole was then initiated in September 2020.

The patient's medical history also included a resected adrenal adenoma in 1991, chronic renal insufficiency (glomerular filtration rate (GFR): 40 ml/min/1.73m^2^), hypertension (treated with atenolol, telmisartan, and hydrochlorothiazide), hypothyroidism, diabetes treated with metformin, hyperuricemia treated with febuxostat, and background of overweight. The family history revealed a mother diagnosed with hypertension with stroke at the age of 60.

She was first admitted to the hospital for transient adrenal insufficiency requiring steroid compensation and hydration. A few days after being discharged, our patient presented prodromal headaches followed by a generalized seizure witnessed by the nurse doing blood sampling at home. After a gradual return to a normal vigilance state, she was driven to the emergency room for diagnosis and treatment. She had no fever with slight tachycardia (120 bpm) and elevated blood pressure (179/101 mmHg) with a normal glucose level. Rapidly following hospital admission, she developed a second occurrence of a generalized convulsive seizure. At the time of consciousness recovery, she remained oriented, consistent, and responding to simple commands. There were neither focal neurologic signs nor symptoms of intracranial hypertension.

No further event occurred when the patient was treated with intravenous clonazepam, followed by oral levetiracetam (500 mg bid). Anti-hypertensive drugs were resumed (with replacement of hydrochlorothiazide by amlodipine), and fludrocortisone was discontinued. Palbociclib was permanently discontinued for the treatment of breast cancer.

**Figure 1:**
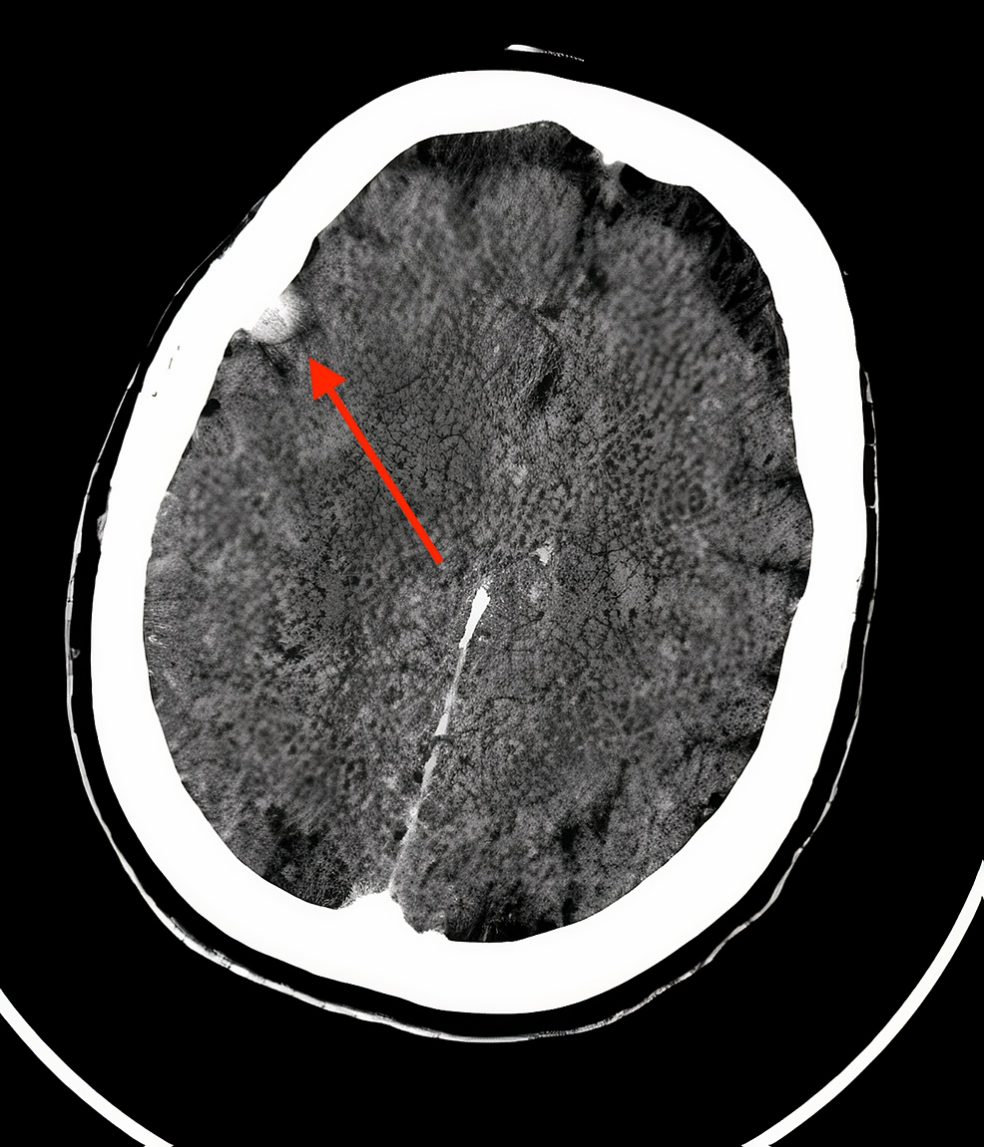
Contrast cerebral computerized tomography scan A small extra-axial right frontal lesion, enhancing vividly, strongly suggestive of meningioma

The contrast cerebral CT scan performed at the entry in the emergency care unit found a small extra-axial right frontal lesion, enhancing vividly, strongly suggestive of meningioma. There was no obvious focal parenchymal lesion (Figure 1).

**Figure 2:**
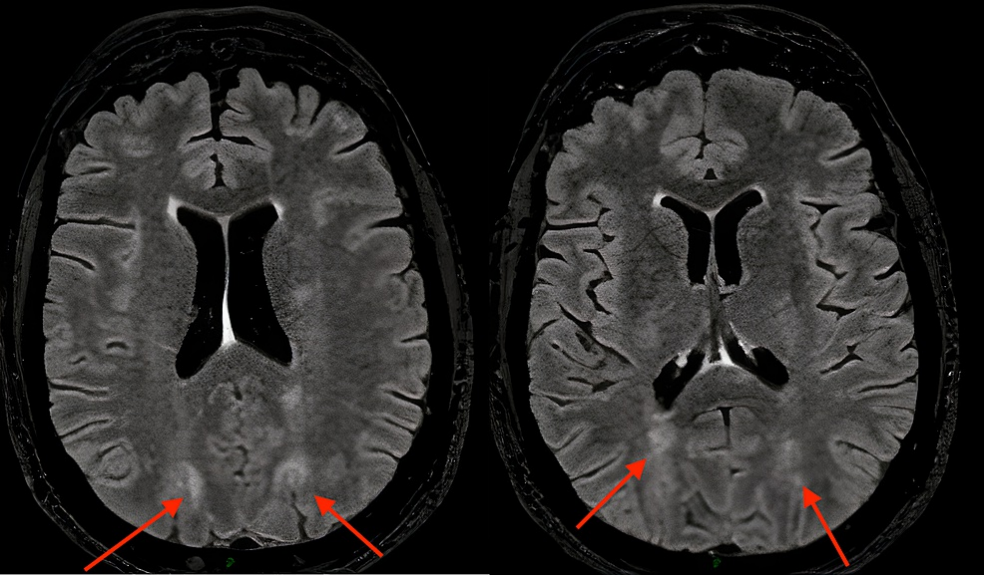
Fluid-attenuated inversion recovery (FLAIR) magnetic resonance imaging Bilateral, globally symmetrical, sub-cortical parieto-occipital FLAIR hypersignals of white matter

**Figure 3:**
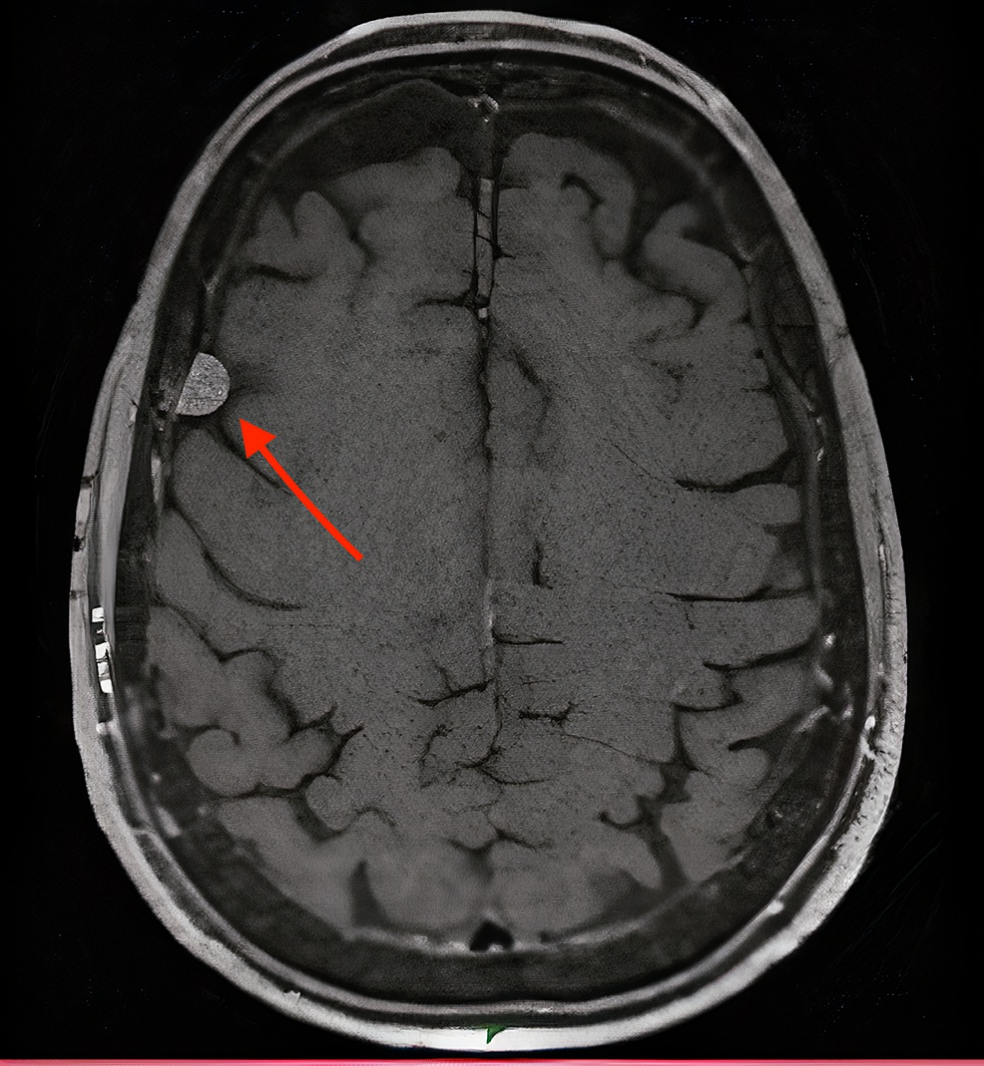
Gadolinium-enhanced T1-weighted magnetic resonance imaging Right frontal extra-axial formation: the size was 9 mm and there was no mass effect

The MRI found a bilateral, globally symmetrical, sub-cortical parietooccipital fluid-attenuated inversion recovery (FLAIR) hypersignal of the white matter. This hypersignal was slightly extending to the posterior temporal lobes. There were also some focal FLAIR hypersignals of the supra and subtentorial white matter. The cerebral perfusion was generally symmetrical with a slight bilateral posterior hyperperfusion. A right frontal extra-axial formation was found: centimetric, with a large meningeal implantation base, and intensely enhanced after injection in a homogeneous manner (Figures 2-3).

The context of the recent hypertensive crisis (recent discontinuation of anti-hypertensive therapies for an episode of acute adrenal insufficiency and treatment with fludrocortisone), prodromal headaches, and MRI aspects were consistent with a PRES syndrome. No anti-neuronal antibodies among NMDA, Yo, Hu, Ri, LGI1, and Caspr2 were found.

**Figure 4:**
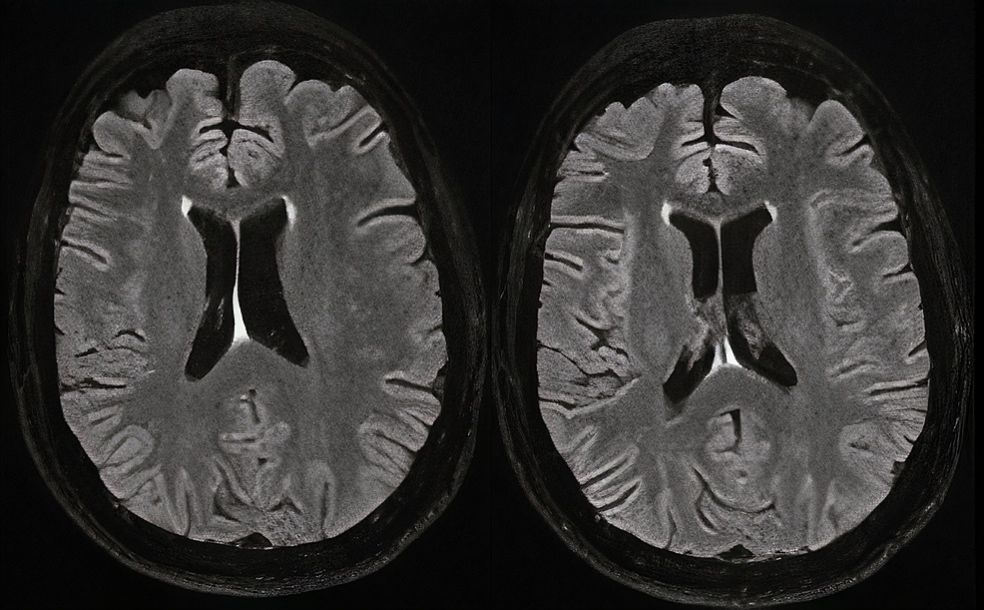
Fluid-attenuated inversion recovery (FLAIR) magnetic resonance imaging performed one month later Total regression of the FLAIR signal abnormalities

Subsequently, follow-up using MRI one month later showed a total regression of the FLAIR signal abnormalities (Figure 4). There had been no recurrence of the seizure or sequelae and treatment with levetiracetam was well-tolerated.

## Discussion

This clinical and radiological presentation raises the question of the CDK4/6 inhibitor's possible causative role, which remains difficult to assess. It is obvious that this single case doesn't allow any definitive conclusion to be drawn. However, the imputability appeared sufficient for our neurology multidisciplinary consultation to decide to stop the treatment. The same decision was taken in a similar presentation reported in the literature.

Indeed, to our knowledge, this is the third published case of PRES occurring during treatment with palbociclib.

The 2019 case reported by Harrold et al. involved a 56-year-old woman undergoing second-line treatment with palbociclib and fulvestrant [5]. She was admitted with progressive headache and dizziness and was normotensive. The neurology multidisciplinary consultation meeting considered that palbociclib was probably the causative agent and discontinuation of this treatment without a subsequent attempt of reintroduction had been decided.

The case reported by Zappia et al. was of a 60-year-old patient who had previously received several lines of chemotherapy/targeted agent, including bevacizumab, and was being treated with palbociclib and fulvestrant [6]. She was also normotensive when she presented with neurological symptoms. The other factors that may have contributed to this occurrence of PRES were brain radiation therapy six months earlier, cerebral thrombosis, and magnesemia at the lower limit of normal. The episode resolved quickly after treatment with magnesium sulfate, dexamethasone, and mannitol. Treatment with palbociclib and fulvestrant was discontinued.

In our case, the unexpected diagnosis of concomitant meningioma was considered as a differential diagnosis for the occurrence of convulsive seizures. However, given its small size (9 mm), together with the absence of peritumoral edema and mass effect, it was considered unlikely that meningioma would be the only factor that could have induced seizure. Moreover, none of the other known risk factors of seizure in supratentorial meningiomas, such as male gender, absence of headache, non-skull base location, were involved [7]. 

Another potential differential diagnosis or contributing factor is the use of corticosteroids for an episode of adrenal insufficiency, as there are reports incriminating them as a trigger for PRES [8]. However, this has been described for high-dose corticosteroids administration (2 mg/kg equivalent prednisone dose per day), whereas our patient required only 0.2 mg/kg equivalent prednisone dose.

The fludrocortisone dosage was 50 micrograms per day (not increased for the acute episode) and may have been a contributing factor, as mineralocorticoid excess is known to promote hypertension and has already been reported to be associated with PRES [9].

To our knowledge, no other drug the patient was taking is reported to be connected to PRES. The recent discontinuation of antihypertensive therapies, though, was probably another contributing factor.

The mechanism by which palbociclib could trigger the syndrome would be largely uncertain. It may be a vascular endothelium dysfunction induced by the cytotoxic effect of the molecule, as well as the stimulation of an inflammatory cascade and/or hypomagnesemia, as suggested in the case reported by Zapia et al. In fact, in pre-clinical and clinical studies, 13%-20% of patients treated with combination palbociclib-fulvestrant presented with hypomagnesemia [6]. In the case presented here, magnesemia had been measured at 0.62 mmol/L 10 days before the symptom’s occurrence and at 0.52 mmol/L 15 days before it (standard: 0.66 to 1.07 mmol/L), and this hypothesis, therefore, seems interesting to us to explore.

## Conclusions

This case report aims to encourage physicians whose patients are treated with cyclin-dependent kinase 4/6 inhibitors to cautiously monitor symptoms suggesting PRES in contexts known to promote its occurrence such as that of arterial hypertension, immunosuppression, and/or autoimmune disease. PRES should be considered in the event of seizure, headache, and/or visual disturbances.
